# Effects of Mobile Application Program (App)-Assisted Health Education on Preventive Behaviors and Cancer Literacy among Women with Cervical Intraepithelial Neoplasia

**DOI:** 10.3390/ijerph182111603

**Published:** 2021-11-04

**Authors:** Yi-Hui Lee, Lian-Hua Huang, Su-Hui Chen, Jung-Hua Shao, Chyong-Huey Lai, Nan-Ping Yang

**Affiliations:** 1 School of Nursing, College of Medicine, Chang-Gang University, Taoyuan 333323, Taiwan; yhlee@mail.cgu.edu.tw (Y.-H.L.); shao@mail.cgu.edu.tw (J.-H.S.); 2 School of Nursing, China Medical University, Taichung 404333, Taiwan; lhhuang@ntu.edu.tw; 3 School of Nursing, Chang Gung University of Science and Technology, Taoyuan 333324, Taiwan; sophee@gw.cgust.edu.tw; 4 Department of Rheumatology, Allergy, and Immunology, Chang Gung Memorial Hospital, Linkou Branch, Taoyuan 333423, Taiwan; 5 Department of Joint Reconstruction, Chang Gung Memorial Hospital, Linkou Branch, Taoyuan 333423, Taiwan; 6 Department of Obstetrics and Gynecology, Chang Gung Memorial Hospital, Linkou Branch, Taoyuan 333423, Taiwan; 7 Gynecologic Cancer Research Center, Chang Gung Memorial Hospital, Linkou Branch, Taoyuan 333423, Taiwan; 8 School of Medicine, College of Medicine, Chang-Gang University, Taoyuan 333323, Taiwan; 9 Community Medicine Research Center, National Yang-Ming Chiao Tung University, Taipei 112304, Taiwan; 10 Department of Medical Research, Hualien Hospital, Ministry of Health and Welfare, Hualien 970007, Taiwan

**Keywords:** health education, application program (App), supportive technology, innovative model

## Abstract

Objective: This study aimed (1) to study the effects of health education on preventive behaviors and cancer literacy among women with cervical intraepithelial neoplasia (CIN); (2) to compare the effects of mobile application program (App)-assisted health education with traditional book-form health education. Participants: A total of 132 women ages 20 to 69 years women. Methods: This prospective longitudinal study enrolled 132 CIN women who were evaluated three times. Propensity score matching was used by controlling subjects’ age strata, body mass index, education level, occupation, and type of surgery. Results: The influences of various educational tools were investigated. Four domains were assessed, including health behavior, attitude towards behavior change, self-efficacy of behavior, and cervical cancer (CCa) literacy. Significant improvements in behavior change and CCa literacy due to a health education program were observed (*p* ≤ 0.002). The App combined with a traditional booklet had the highest score for behavior change and was significantly greater than the booklet-only learning (*p* = 0.002). The App-assisted form, either App alone or combined with booklet, had a significantly better impact on health promotion when compared to the booklet alone (*p* = 0.045 and 0.005, respectively). App-only learning had the highest score of CCa literacy (*p* = 0.004). Conclusion: Health education interventions can have positive effects in terms of change of behavior and CCa literacy. App-assisted learning could be used as a supportive technology, and App learning alone or combined with a traditional booklet may be an innovative model of clinical health promotion for women with CIN.

## 1. Introduction

For females, a cancer diagnosis might provide a teachable moment for health behavior change in the period immediately following diagnosis [1]. Furthermore, health education interventions were significant in terms of improving knowledge and perceptions and increasing the self-efficacy of women with regards to cervical cancer and screening [2]; those with inadequate health literacy were more likely to undergo irregular cervical cancer screening [3], and married women’s knowledge levels about cervical cancer and Pap smear screening were increased as their self-efficacy level, and health literacy level increased [4]. In Taiwan, a cervical cancer screening program for women over 30 years of age, who are eligible for one free Pap smear annually, had decreased 69.5% age-standardized incidence rate of invasive cervical cancer and 70.9% age-standardized mortality rate of cervical cancer since 1995, but the screening coverage was only 50.4% in 2019 [5]. Women with inadequate health literacy tended to not follow the Pap smear routine screening [3]. In Taiwan, the coverage rate of National Health insurance (NHI) was 99.9%, and the universal service coverage index (SCI) was 87.0% which was only lower than Canada according to the WHO report [6]. The challenges of increasing the cervical screening rate would be how to facilitate health education and literacy of cervical cancer, provide screening reminders, and deliver reports [7,8], especially in areas without digital cervicography reading or with inadequate health worker training [9].

Mobile health services are a supportive technique that has gradually been introduced to support patients in their self-management of chronic disorders, such as hypertension [10], cardiovascular diseases [11], diabetes [12], and obesity [13], and even improve patients’ medication adherence [14]. Recently, some smartphone apps were designed to address the COVID-19 pandemic [15], while an evidence-based mobile health (mHealth) program led to significant reductions in depressive symptoms [16]. For some disease-specific populations, the aggressive use of health-related applications (Apps) on mobile phones could be useful for clinical health promotion. In line with the current trend for care to be provided nearer patients’ homes, telephone interventions have been demonstrated to provide a convenient way of supporting self-management of cancer-related symptoms for adults with cancer; this could be augmented by combining telephone interventions with face-to-face meetings and the provision of printed or digital materials [17]. A previous study reported that people appreciated the timeliness and convenience of mHealth [18] but resisted change [19]. It was very important to establish an effective and convenient health education method to help those newly diagnosed patients. In the past, a rare study of mHealth education was performed to compare the effectiveness between different tools. An orthopedics study indicated that using an App and a booklet with the same health education context led to similar cost-effectiveness ratios and the same occurrence of recurrent rates [20]. A recent review pointed that mHealth intervention was effective in increasing participants’ knowledge of cervical cancer and screening rate in clinics, but the evidence of its effectiveness was not comprehensive [21].

Traditionally, health education or health promotion is considered an asset within health care and can provide relevant information to subjects or groups to prevent disease or improve its consequences. Health literacy could be defined as the ability of citizens to meet the complex demands of health in modern society. Self-efficacy may also be seen as confidence in one’s ability to perform well in a domain of life that may be associated with improved health behavior or a decreased disease burden. Patients with various cancers may benefit from the support of an App-based health promotion program in terms of advancing health literacy [22], enhancing related behavior changes, such as self-efficacy and self-management [23], reducing nursing care needs, and improving quality of life after surgery [24]. App-assisted health education is an innovative model used to increase health literacy and improve health-related behaviors, including self-efficacy of behavior and positive behavioral change, and may be useful in some cancer patients.

Therefore, this study enrolled women with cervical intraepithelial neoplasia (CIN); it aimed to examine the effects of health education on preventive behaviors and cancer literacy and to compare the effects of App-assisted health education with those of traditional book-form health education.

## 2. Materials and Methods

This prospective longitudinal study was conducted in the gynecology outpatient of a medical center with 4000 acute beds in North Taiwan. Women with CIN cytology of Pap smear within 3 months were recruited through face-to-face contact and, the informed consent and self-reported questionnaire were provided after obtaining their permission. The present study was classified as a convenience sampling study that was based on the Transtheoretical Model using a questionnaire to investigate the studied patients’ health behavior (including health promotion, risk control and cancer prevention), the attitude of health behavior, self-efficacy of health behavior, and cancer literacy

### 2.1. Study Population and Protocol

**Figure 1:**
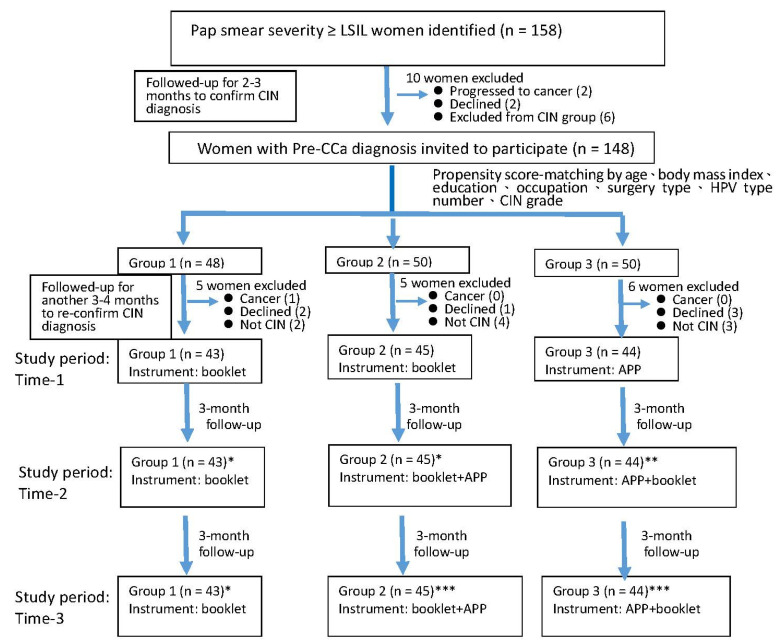
Enrolled subjects and study protocol. Abbreviations: LSIL, low-grade squamous intraepithelial lesion; HPV, human papillomavirus; CIN, cervical intraepithelial neoplasia. Notes: * actual effect of the booklet-form instrument; ** actual effect of the App-form instrument; *** actual effect of the App plus booklet-form instrument.

Initially, this study enrolled 158 gynecological outpatients with newly diagnosed cervical dysplasia (with a severity greater than or equal to the low-grade squamous intraepithelial lesion (LSIL)) following a Pap smear at a medical center in North Taiwan. Because diagnostic procedures took around 6 months for an LSIL to be confirmed of histology of CIN [25,26], all the targeted patients were followed for 6 months before enrollment. Before the study period, women having one CIN cytology report or atypical squamous cells of undetermined significance (ASCUS) report would be excluded from a cancer situation. Finally, all the targeted women had more than two cytology or histology CIN reports, and there were 148 patients included in the next stage (see Figure 1).

In the previous study, a multiple propensity score was used to reduce bias in nonrandomized studies as an effective tool [27]. In the present study, biostatistical software was used to perform the multiple propensity score [28]. First, all the socio-economic variables and gynecological evaluations (such as Pap smear result, CIN grade) of 148 candidates were collected and calculated by a multiple logistic regression analysis. Second, the propensity score matching method was used, and their suggested matching IDs were produced by the biostatistical software. Third, the suggested matching IDs were double-checked to prove the consistency of grouping and complete the matching process. The 148 candidates who were invited and agreed to attend this study were arranged into three groups based on their multiple propensity scores matched by controlling subjects’ age strata, body mass index classification, education level, occasion status, and type of surgery. There was 1 participant who progressed to CCa, 6 participants declined, and 9 participants were excluded CIN due to only one abnormal Pap smear result. At the end of the follow-up period, 132 women with a defined CIN participated in the subsequent part of the study (see Figure 1).

Subsequently, all the study subjects were assessed three times: at baseline (Time-1) and every 3 months thereafter (Time-2 and Time-3) for 6 months in total. The subjects were provided with various types of educational tools after every measurement. Those in the first group were offered booklet-form education materials during the 6-month study period; after the study had finished, they were also provided with the App-form instrument. Those in the second group were offered the educational booklet at Time-1, then App-form education at Time-2, while those in the third group were offered App-form learning at Time-1, then the booklet at Time-2. The effects of the various forms of health education were evaluated by repeated measurements as compared with the outcomes at Time-1. The effects of the mobile App-assisted education model were evaluated and simulated using the generalized linear statistics (see Figure 1).

### 2.2. Health Education Tools

Prior to the present study, 10 women with a new CIN diagnosis were interviewed to assess their health education needs in a pilot survey during the pre-test period. Eighteen questions were developed, and suitable answers were established based on publicly-available information from the National Health Research Institute (Taiwan), National Health Promotion Administration (Taiwan), Ministry of Health and Welfare (Taiwan), World Health Organization (WHO), National Cancer Institute (USA), and recent articles in high impact-factor gynecological journals. The content of the CIN health education included the definition of CIN, the meaning of the Pap smear result, the need for regular examination or surgery, the CIN follow-up procedure, common cancer prevention heath behaviors (e.g., cancer screening, *human papillomavirus* (HPV) vaccine, HPV screening), cervical cancer prevention and survival, risk-control health behaviors related to CIN (e.g., cessation of smoking and drinking alcohol), and health promotion behaviors relative to CIN (e.g., physical activity, balanced diet, bodyweight control). All content was included in the booklet-form educational material.

**Figure 2:**
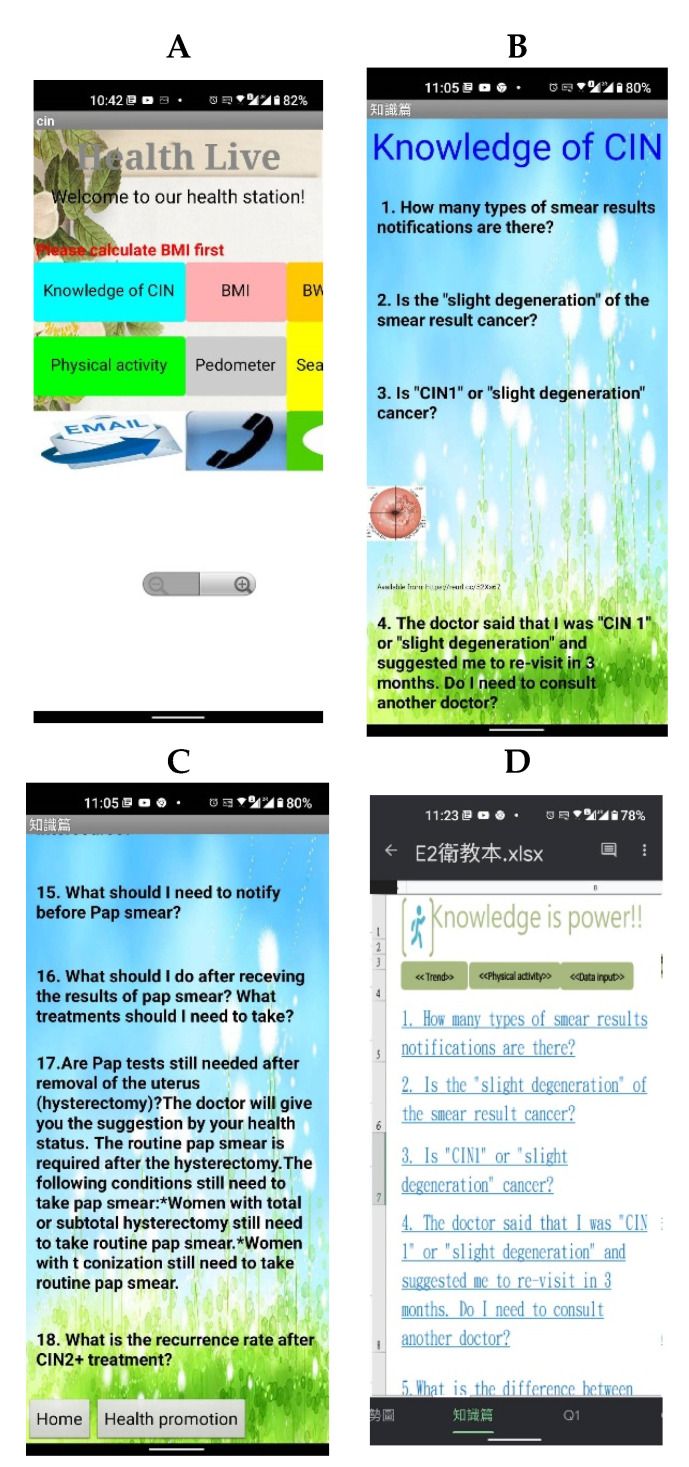
Photos of App screen, translated into English (initial in Chinese) ((**A**) home page, (**B**) first page of health education, (**C**) 12th to 18th questions as an example; 17th and 18th answers listed if question selected, (**D**) alternative EXCEL form provided).

Considering the requirements of the enrolled women, two different kinds of mobile educational material were used: (1) CIN health education App containing all the contents included in the booklet (see Figure 2A–C) and (2) alternative EXCEL files containing the same educational information, which was needed due to limitations of some cellphones (see Figure 2D).

### 2.3. Instruments for the Assessment of Three Health Behavior-Related Domains and One Literacy Domain

#### 2.3.1. Health Behavior Instrument

Based on the Trans-Theoretical Model (TTM), a 14-item questionnaire (Supplementary Materials) was designed to assess health behavior, which was divided into three subscales according to the results of factor analysis: health-promotion behaviors, risk-control behaviors (i.e., health hazards), and cancer-prevention behaviors. The internal consistency reliability, Cronbach’s α, of this instrument was 0.70, the content validity was 0.99, and the test-retest reliability was 0.91, as calculated during the 6-month follow-up period.

#### 2.3.2. Health Behavior Change Instrument

A 15-item questionnaire (Supplementary Materials), consisting of the 14 items in the health behavior instrument and an additional HPV vaccine item (classified into the cancer-prevention subscale), was designed to detect health behavior change and was also divided into three subscales. The internal consistency reliability, Cronbach’s α, of this instrument was 0.86, the content validity was 0.93, and the test-retest reliability was 0.89, as calculated during the 6-month follow-up period.

#### 2.3.3. Self-Efficacy of Health Behavior Instrument

A 10-item questionnaire (Supplementary Materials) was designed to detect the self-efficacy of health behavior and was also divided into three subscales. The internal consistency reliability, Cronbach’s α, of this instrument was 0.79, the content validity was 0.98, and the test-retest reliability was 0.88, as calculated during the 6-month follow-up period.

#### 2.3.4. CCa Literacy Instrument

A 10-item questionnaire was designed to detect CCa literacy from one general score according to the experts’ discussion and conclusion. The internal consistency reliability, Cronbach’s α, of this instrument was 0.65, the content validity was 0.99, and the test-retest reliability was 0.75, as calculated during the 6-month follow-up period.

### 2.4. Statistical Analysis

The data are presented as case number, percentage, mean, and standard deviation (SD). Socio-economic and descriptive data were compared between the three groups using the chi-square test and one-way analysis of variance (ANOVA). Factor analysis was performed to condense multiple items into fewer subscales. To evaluate the trends in domains or subscales, repeated-measure ANOVA was performed, and the results were adjusted by the Greenhouse-Geisser (G-G) method; the post-hoc test was also applied. Propensity score matching was used to match several possible confounders among groups, and a generalized linear model (GLM) was used to simulate the effects of the App-assisted educational model. Statistical analyses were performed using the program SPSS 24, and multiple propensity score was performed by using SAS 9.4 and SPSS 24.

## 3. Results

### 3.1. Effects of Grouping by Propensity Score Matching

In total, 132 women with newly diagnosed CIN were enrolled in the present study; who had a mean age of 46.9 (SD, 12.4). All studied women were arranged into three groups by propensity score matching. There were no significant differences in terms of age strata, education level, occupation, socio-economic status, marital status, chronic disease status, body mass index (BMI), CIN grade, or a number of HPV types between groups. Of the enrolled women, 76.5% underwent various types of surgery, and undergoing surgery could influence the reporting of pain, depression, negative feelings, and sexual discomfort, which might subsequently decrease the patient’s self-efficacy to improve their health behavior (see Table 1).

### 3.2. Effects of Health Education

The health behavior score and self-efficacy of health behavior score slowly increased from baseline to the third measurement (28.6 to 29.5 and 32.9 to 33.4, respectively), but there were no significant differences between groups (*p* > 0.05). After health education intervention, the health behavior change score significantly increased following the introduction of learning programs (20.0, 25.6 and 26.2, respectively, *p* = 0.002), which demonstrated an improved attitude towards changing health behavior, in particular in terms of health promotion and cancer prevention (with an increasing trend in the scores of both subscales, *p* = 0.005, 0.018, respectively). Similarly, the CCa literacy score was also significantly increased following the introduction of education programs (6.6, 7.4 and 7.4, respectively, *p* < 0.0001). In addition, in terms of the concept of risk control, which falls within the subscale of self-efficacy of behavior, an improved result was obtained due to health education (*p* = 0.016) (see Table 2).

### 3.3. Effects of a Mobile App-Assisted Education Model

**Figure 3:**
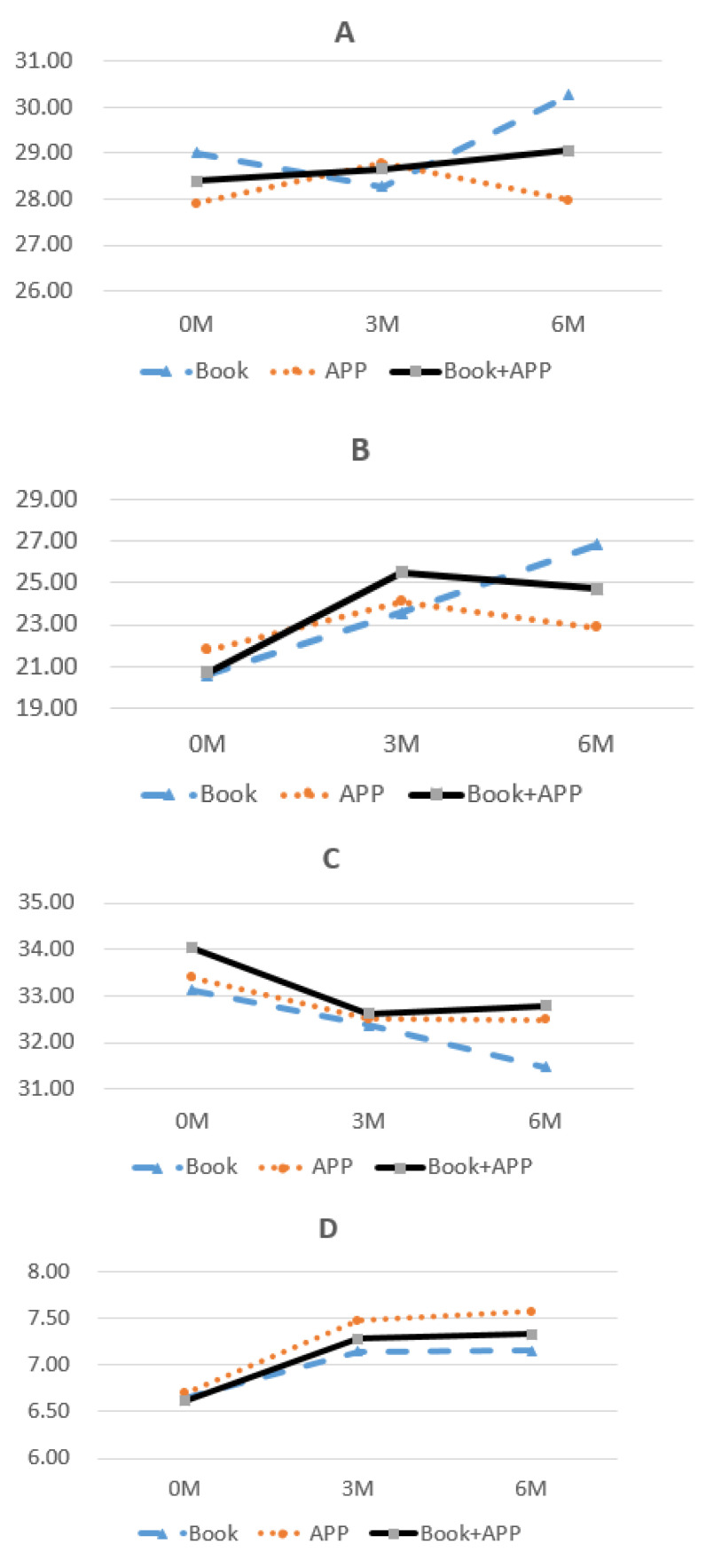
Simulated trends of four domains by time ((**A**) Health behavior; (**B**) Attitude towards behavior change; (**C**) Self-efficacy of behavior; (**D**) CCa literacy).

Different educational intervention tools were offered to the participants of the present study. Owing to the complex study design, the GLM method was used to simulate and evaluate the effects of the App-assisted educational intervention, based on actual data for the second and third groups at Time-2 and Time-3. Regarding health behavior, the introduction of the App combined with the booklet resulted in a gradually increasing trend (see Figure 3A). Focusing on the effects on attitude towards behavior change and cancer literacy, all three educational models resulted in increasing trends over time (see Figure 3B,D). Conversely, all three models had simulated decreasing trends in self-efficacy over time (see Figure 3C).

GLM analysis was used to investigate the interactions between different measurement times and different tools. At Time-2, the actual difference between booklet-form and App-form learning could be estimated, while at Time-3, the actual difference between booklet-form and App combined with booklet-form learning could also be estimated (see Figure 1). Remarkably, interactions of measurement times and different tool types could be assessed and were significant in the behavior change domain (F = 3.2, *p* = 0.043) and the health promotion subscale (F = 4.5, *p* = 0.012) (see Table 3). In general, the App combined with the booklet resulted in the highest score for attitude towards behavior change, which was significantly greater than the score for booklet-only learning (24.6 vs. 24.1, respectively, *p* = 0.002); App-only learning resulted in the highest score for CCa literacy, which was significantly greater than the score for the booklet-only form (7.4 vs. 7.1, respectively, *p* = 0.004). In particular, for the health promotion concept within the subscale of behavior change, it was noted that App-assisted learning, either alone or combined with traditional booklet learning, had a positive effect as compared with the booklet alone (5.81 and 5.8 vs. 5.8, *p* = 0.045 and 0.005, respectively) (see Table 3).

## 4. Discussion

Cancer-related knowledge and cancer literacy are important for the cancer population; having adequate knowledge results in appropriate actions, whereas a lack of knowledge leads to inappropriate actions [29,30]. Health education interventions were significant in terms of improving CCa literacy, self-efficacy, and taking actual activities [2,3,4,31,32]. Similarly, the present study showed that repeated health education interventions could be valuable and had positive effects on the change of behavior, partial self-efficacy of behavior, and CCa literacy among Taiwanese women with CIN.

A positive experience of using social media related to health might be important in terms of people’s intentions regarding health behavior. A cross-sectional survey of about 450 adults revealed that the association between health-related social media use and self-efficacy was stronger among those who had previously had positive experiences with health information on social media [33]. Based on data from the Health Information National Trends Survey (HINTS), performed in the USA, health-related internet use (HRIU) was evaluated; the results revealed that HRIU varied greatly and significantly by demographics and intended use, and 80% had looked online for health information [34]. In a modern society, social media use must be considered as a tool by which to introduce health education programs. Furthermore, various health-related Apps should be encouraged and developed. Usually, health-related Apps are published on the two leading platforms, iOS and Android.

The effectiveness of mobile phone Apps in achieving health-related behavior change was worthy of further evaluation. The widespread adoption of mobile phones and health-related Apps might highlight a significant opportunity to impact health behavior globally, particularly in low- and middle-income countries [35]; a steady increase in the rigorous evaluation of Apps aims to modify behavior to promote health and manage disease [36]. Moreover, using mHealth Apps as a widespread form of supplementary clinical support showed promise with regards to increasing patient education, narrowing the knowledge gap in health-disparate communities, and even reducing the burden on the care partner [37,38]. Targeted at primary care patients, the cancer prevention mobile App and mHealth program could facilitate behavioral change to reduce the risk of cancer [39]. In the present study, App-assisted mHealth education was provided to patients with CIN and was noted to have positive effects on behavior change and cancer literacy.

A recent survey of 794 oncology Apps in the Apple iOS and Google Play App stores showed that the majority of oncology-specific Apps were free; only 27% were intended for patients, and the intended function was education for 36% followed by clinical decision support for 20% [40]. The mHealth App used in this study was free for all the enrolled patients, who found it satisfactory and helpful. Some features have been noted that could improve the effectiveness of Apps, such as less time consumption, a user-friendly design, real-time feedback, individualized elements, detailed information, and health professional involvement [35]. In future, the App used in this study could be re-designed and updated to provide a much more high-quality service.

Health literacy was noted to be significantly associated with age, health status, and health problems [41]; it is also seen as an indicator by which to describe a nation’s health status. A national health literacy survey, including nearly 9500 individuals aged 18 and above, showed that limited health literacy groups were prevalent among respondents of an older age, a lower education level, and with a lower household income [42]. Another nationwide cross-sectional survey enrolling over 9000 Danish individuals aged ≥ 25 years showed that inadequate health literacy is strongly associated with a low socio-economic status, a poor health status, inactivity, and being overweight [43]. In the present study, CCa literacy was improved by repeated health education programs and was positively associated with App-assisted model use, either alone or combined with the booklet form. However, other potential associated factors were controlled by a matching method, as identification of those factors was not the main goal of this study.

Behavior changes are important for patients with cancer. Cancer survivors were more likely to have made positive than negative behavior changes after cancer; positive changes are correlated with a younger age, greater education level, breast cancer, a longer duration since diagnosis, fear of recurrence, spiritual well-being, etc. [44,45]. Relative to those with single cancers, multiple primary cancer survivors are at increased risk of psychological distress and are more likely to attend the recommended cancer screenings [46]. In the present study, only women with pre-cervical cancerous lesions were enrolled; the attitude towards behavior change was improved by the introduction of health education programs and was positively associated with the implementation of App-based learning combined with the booklet form.

Furthermore, in this study, among the subscale of attitude towards behavior change, cancer prevention and health promotion were also improved by the introduction of health education programs; this may be related to fear of cancer recurrence (FCR), which is highly prevalent among adult survivors of cancer and known to have an impact on both cancer survivors and their caregivers [45,47]. Observed in other studies, FCR was noted to play a central role in the emotional distress and key health behaviors of either survivors of cancer or their caregivers, and also uniquely promoted their engagement in cancer screening behaviors [45,47,48].

A study of a nationally-representative sample of about 4,800 adults aged 65 and older in the USA showed that self-efficacy may be a key modifiable element to incorporate into multimodal physical frailty interventions [49]. A cross-sectional survey including 213 cancer patients revealed that participants with a higher self-efficacy had higher chemotherapy self-management scores [50]. As self-efficacy was known to be inversely related to physical problems [51], 76.51% of the enrolled women received different type of surgeries, and their negative experience might decrease their self-efficacy from Time-1 to Time-2 in the present study. In general, self-efficacy of behavior, especially the concept of risk control, was investigated and was improved due to the educational interventions; the general self-efficacy score was found to be positively correlated with App-assisted model use, either alone or combined with the booklet form, although this was not statistically significant.

## 5. Limitations

A defined CIN diagnosis takes time because a CIN diagnosis and management requires at least 6 months to be confirmed; the women were followed-up in outpatient clinics or outpatient surgery. Some subjects were susceptible to ignoring regular follow-ups or changing to other clinics; therefore, six initially-enrolled subjects were excluded from the study. The number of subjects was limited, and effort was needed to maintain their participation.

Compared with the virtual App, many of the enrolled women prefer a tangible education booklet for feeling more adaptable. Moreover, some of the subjects’ mobile phones were too old or new to allow installation of the App. Alternative interventions were taken using EXCEL files. Therefore, it was difficult to design a pure App-only group in the present study.

The WHO suggested that a cervical cancer program could have five dimensions of mobile-related intervention: mAwareness, mTraining, mQuality-Assurance, mFollow-up, and mSurveilance [21]. This study may be a pilot study of mAwareness and mFollow-up; however, the App used in the present study consisted of multiple interfaces that could not be differentiated by their separate effectiveness that should be studied by a stratification analysis in the future.

## 6. Conclusions

This study was performed among women with pre-cervical cancerous lesions, and the results showed that health education intervention can have a positive effect on preventive behaviors, especially on the change of behavior and CCa literacy. App-assisted health education could be used as a supportive technology; App use alone or combined with traditional booklet learning may be an innovative model of clinical health promotion for women with CIN.

## Figures and Tables

**Table 1:** Basic characteristics of the enrolled subjects (n = 132).

Variable	Group 1 (n = 43)		Group 2 (n = 45)		Group 3 (n = 44)		*p*-Value
	n (%)	Mean ± SD	n (%)	Mean ± SD	n (%)	Mean ± SD	
**Age**		47.1 ± 11.6		46.0 ± 13.2		47.4 ± 12.6	0.864
<35 y/o	7 (16.3)		7 (15.6)		4 (9.1)		0.949
35–49 y/o	18 (41.9)		18 (40.0)		21 (47.7)		
50–64 y/o	15 (34.9)		17 (37.8)		15 (34.1)		
>65 y/o	3 (6.9)		3 (6.7)		4 (9.1)		
**Education level**							
Illiterate	1 (2.3)		2 (4.4)		1 (2.2)		0.951
Primary and middle	10 (23.3)		14 (31.1)		15 (34.1)		
High school	22 (51.2)		18 (40.0)		19 (43.2)		
Bachelor	9 (20.9)		9 (20.0)		7 (15.9)		
Graduate	1 (2.3)		2 (4.4)		2 (4.6)		
**Occupation**							
Manager	1 (2.3)		1 (2.2)		1 (2.3)		0.925
Professional	11 (25.6)		8 (17.8)		7 (15.9)		
Technician	5 (11.6)		6 (13.3)		3 (6.8)		
Technical staff	6 (14.0)		7 (15.6)		6 (13.6)		
Semi-technical staff	20 (46.6)		23 (51.1)		27 (61.4)		
**Socio-economic status**							
I, II	12 (27.9)		12 (26.7)		14 (31.8)		0.596
III	22 (51.2)		24 (53.3)		26 (59.1)		
IV, V	9 (20.9)		9 (20.0)		4 (9.1)		
**Marital status**							
Single	1 (2.3)		4 (8.9)		2 (4.5)		0.790
Couple	4 (9.3)		5 (11.1)		5 (11.4)		
Married	32 (74.4)		27 (60.0)		30 (63.2)		
**Body mass index**		23.4 ± 3.4		22.6 ± 3.0		23.8 ± 4.0	0.268
Underweight	2 (4.7)		3 (6.7)		2 (4.6)		0.827
Normal	21 (48.8)		27 (60.0)		20 (45.5)		
Overweight	12 (27.9)		8 (17.8)		12 (27.3)		
Obese I	5 (11.6)		6 (13.3)		6 (13.6)		
Obese II, III	3 (7.0)		1 (2.2)		4 (9.1)		
**No. of chronic diseases**		0.4 ± 0.9		0.4 ± 0.7		0.5 ± 0.6	0.958
**CCI index**		0.4 ± 0.9		0.4 ± 0.7		0.5 ± 0.6	0.816
**Smoking everyday**							
Never	37 (86.0)		38 (84.4)		37 (84.1)		0.510
Sometime	0 (0.0)		3 (6.7)		2 (4.5)		
Usually	1 (2.3)		0 (0.0)		2 (4.5)		
Always	5 (11.6)		4 (8.9)		3 (6.8)		
**The most serious of CIN grade (cytology)** **during the study period**							
Mild dysplasia (CIN I)	13 (30.2)		10 (22.2)		14 (31.8)		0.245
HSIL dysplasia	13 (30.2)		10 (22.2)		16 (36.4)		
Severe dysplasia	17 (39.5)		25 (55.6)		14 (31.8)		
**The most serious of CIN grade (histology)** **during the study period**							
CIN 0	4 (9.3)		1 (2.2)		2 (4.5)		0.477
CIN 1	11 (25.6)		9 (20.0)		15 (34.1)		
CIN 2	17 (39.5)		20 (44.4)		13 (29.5)		
CIN 3^+^	11 (25.6)		15 (33.3)		14 (31.8)		
**Risk of HPV type**							
No test	6 (14.0)		4 (8.9)		6 (13.6))		0.505
None of HPV	10 (23.3)		5 (11.1))		8 (18.2)		
lLw-risk HPV type	5 (11.6)		11 (24.5)		10 (22.7)		
High-risk HPV type	18 (41.9)		18 (40.0)		14 (31.8)		
Type 16 or 18	4 (51.2)		7 (55.6)		6 (13.6)		
**Surgery type**							
No surgery	13 (30.2)		9 (20.0)		9 (20.5)		0.423
Conization	2 (4.7)		2 (4.4)		3 (6.8)		
CO2 laser	6 (14.0)		9 (20.0)		6 (13.6)		
LEEP	22 (51.2)		25 (55.6)		26 (54.6)		

Abbreviations: CCI, Charlson Comorbidity Index; LEEP, loop electrosurgical excision procedure.

**Table 2:** Trends of four measurements in three behavior-related domains with three subscales and one cancer literacy domain.

GLM Method Application	Measurement at Each Time			F-Value * p*-Value (GreenHouse -Geissrer Adjusted)		Post-Hoc (Tukey-Kramer)
	TIME-1, Mean ± SD	TIME-2, Mean ± SD	TIME-3, Mean ± SD			
Health behavior	28.6 ± 7.0	28.5 ± 7.2	29.5 ± 6.2	0.8	0.442	NS
Health promotion	7.8 ± 2.6	8.1 ± 2.8	8.1 ± 2.2	0.7	0.513	NS
Risk control	10.4 ± 2.6	10.2 ± 2.7	10.1 ± 2.1	0.5	0.636	NS
Cancer prevention	10.4 ± 5.0	10.2 ± 5.0	11.3 ± 4.8	1.9	0.15	NS
Attitude towards behavior change	20.0 ± 15.5	25.6 ± 19.1	26.2 ± 16.8	6.5	0.002 **	Time 1 < Time 2, 3
Health promotion	4.3 ± 6.6	6.5 ± 7.9	6.8 ± 7.0	5.6	0.005 **	Time 1 < Time 2, 3
Risk control	6.3 ± 6.3	8.1 ± 8.8	7.7 ± 7.6	1.6	0.154	NS
Cancer prevention	9.5 ± 7.0	11.1 ± 6.9	11.7 ± 7.3	4.1	0.018 *	Time 1 < Time 2, 3
Self-efficacy of behavior	32.9 ± 7.4	32.3 ± 8.2	33.4 ± 5.8	0.4	0.547	NS
Health promotion	15.2 ± 0.4	15.0 ± 0.4	15.1 ± 2.2	0.9	0.911	NS
Risk control	10.3 ± 3.1	10.3 ± 2.9	11.1 ± 1.6	4.3	0.016 *	Time 1 < Time 2, 3
Cancer prevention	7.3 ± 1.4	7.0 ± 1.7	7.2 ± 1.3	1.4	0.259	NS
CCa literacy	6.6 ± 1.9	7.4 ± 1.5	7.4 ± 1.8	12.5	<0.0001 ***	Time 1 < Time 2, 3

Notes: * *p* < 0.05; ** *p* < 0.01; *** *p* < 0.001.

**Table 3:** Comparison of effects of different tools on four measured domains (interaction adjusted by the GLM method).

GLM Model	Mean ± SD (n = 132)	Interaction						Comparison of Different Tools						
		Time * Intervention		Time (1,2) * Intervention		Time (1,3) * Intervention		Tool (B) # vs. Tool (A)				Tool (B) # vs. Tool (A + B)		
		F	*p*	F	*p*	F	*p*	Mean ± SD ^B^	Mean ± SD ^A^	F	*p*	Mean ± SD ^A+B^	F	*p*
Health behavior	28.7 ± 0.4	1.0	0.366	0.5	0.487	0.5	0.588	29.2 ± 0.5	28.2 ± 0.7	1.2	0.270	28.7 ± 0.4	0.4	0.520
Health promotion	8.1 ± 0.1	0.2	0.835	0.3	0.588	0.3	0.752	8.2 ± 0.25	7.9 ± 0.2	0.8	0.384	7.9 ± 0.2	0.8	0.366
Risk control	10.2 ± 0.2	1.6	0.189	0.0	0.970	0.3	0.760	10.4 ± 0.2	9.9 ± 0.2	2.0	0.157	10.1 ± 0.2	0.7	0.391
Cancer prevention	7.1 ± 0.3	0.5	0.603	0.5	0.476	0.7	0.511	10.6 ± 0.5	10.5 ± 0.5	0.2	0.674	10.6 ± 0.3	0.0	0.965
Attitude towards behavior change	24.0 ± 1.4	2.7	0.070	0.0	0.867	3.2	0.043 *	24.1 ± 1. 7	22.9 ± 13.5	2.3	0.081	24.6 ± 2.5	2.3	0.002 **
Health promotion	5.8 ± 0.6	1.4	0.256	0.1	0.705	4.5	0.012 *	5.8 ± 0.9	5.8 ± 1.0	2.7	0.045 *	5.8 ± 0.7	2.8	0.005 **
Risk control	7.5 ± 0.6	3.1	0.049 *	0.0	0.294	2.8	0.064	7.1 ± 1.0	7.9 ± 1.1	2.2	0.087	7.6 ± 0.7	2.1	0.037 *
Cancer prevention	10.8 ± 0.6	1.9	0.178	0.0	0.919	2.2	0.145	10.8 ± 0.9	10.3 ± 1.0	0.8	0.482	10.8 ± 0.6	0.0	0.839
Self-efficacy of behavior	32.9 ± 0.6	0.9	0.398	0.0	0.865	1.8	0.178	32.3 ± 1.0	33.0 ± 1.1	1.0	0.395	33.1 ± 0.7	0.7	0.550
Health promotion	15.1 ± 0.4	1.4	0.262	0.1	0.790	2.7	0.106	15.1 ± 0.6	15.2 ± 0.6	1.3	0.264	15.2 ± 0.4	0.0	0.835
Risk control	10.6 ± 0.2	0.3	0.781	0.0	0.940	0.0	0.863	10.3 ± 0.4	10.6 ± 0.4	0.4	0.773	10.7 ± 0.3	0.2	0.810
Cancer prevention	7.2 ± 0.1	0.2	0.322	0.0	0.946	0.3	0.756	7.0 ± 0.2	7.3 ± 0.2	0.4	0.770	7.3 ± 0.1	0.3	0.610
CCa literacy	7.2 ± 0.1	5.4	0.004 **	0.0	0.958	1.7	0.181	7.1 ± 0.02	7.4 ± 0.2	4.5	0.004 **	7.2 ± 0.2	1.1	0.353

Note 1: * *p* < 0.05; ** *p* < 0.01; #: reference. Note 2: Tool A = App only, Tool B = Booklet only, Tool A + B = App and Booklet.

## Data Availability

The data presented in this study are available on request from the corresponding author and the first author.

## Supplementary Materials

The following are available online at https://www.mdpi.com/article/10.3390/ijerph182111603/s1, The formal questionnaire.
