# Supplementary material for: Effects of Mobile Application Program (App)-Assisted Health Education on Preventive Behaviors and Cancer Literacy among Women with Cervical Intraepithelial Neoplasia
Source: Int J Environ Res Public Health. 2021 Nov 4;18(21):11603. doi: 10.3390/ijerph182111603 (PMC8582743; doi:10.3390/ijerph182111603)
Supplement: Supplementary file 1 [file ijerph-18-11603-s001.zip › ijerph-1418123-supplementary.pdf]

Appendix One: Formal questionnaire

Coding:

(A) Practice of Health behavior

Note. During the **past 4 weeks**, how MUCH health behavior that you have done in your impression?

- **Never(0)**： Never been done, less than 10% do it (0-10%)
- **Sometimes(1)**：Sometimes been done, about 30% to 50% do it (11-50%)。
- **Often(2)**：Most of the time, about 70% or more do it. (51-70%)。
- **Always(3)**： Almost do it, about 80% to 90% or more do it, (71-100%)。

	0 never	1 sometimes	2 often	3 always
1. Control Bodyweight to avoid mild obesity (BMI > 27)	☐	☐	☐	☐
2. Choose a diet with low fat, saturated fatty acids and cholesterol (e.g. Eat less whole milk, red meat, fried food, potato chips or fried food, fast food, fatty meat, chicken skin, and less lard oil and butter to cooking)	☐	☐	☐	☐
3. Choose a diet with less processed meat (less bacon, sausage, ham, bacon, jerky, etc.)	☐	☐	☐	☐
4. Eat 3-5 servings of fruits and vegetables a day<br>● 1 serving : 1/2 bowl cooked vegetables, 1 bowl lettuce,1 orange, 1/3 guavas, 1/4 grapefruit,1/4 papaya, 2 lotus mist, 1 star fruit	☐	☐	☐	☐
5. Get at least 150 minutes of moderate aerobic activity (brisk walking, climbing, car washing, mopping, fitness exercises, etc.) or 75 minutes **of** vigorous aerobic activity(running, climbing stairs, playing ball, etc.) a week.	☐	☐	☐	☐
6. Drink less than 2 servings a day (1 serving:1 can of beer, 1 goblet of red wine or mixed wine, 1 cup of 50cc whiskey) 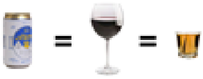	☐	☐	☐	☐
7. Smoking every day	☐	☐	☐	☐
8. Smoking second-hand smoke every day	☐	☐	☐	☐
9. Regular Pap smears (once every 3 years after first time intercourse during 20-30 y/o , or once a year ≧30 y/o)	☐	☐	☐	☐
10. Regular breast screening (breast self-examination after menstruation, or mammography every 2 years≧45 y/o)	☐	☐	☐	☐
11. Regular oral cancer screening (oral mucosal examination once every 2 years)	☐	☐	☐	☐
12. Regular colorectal cancer screening (fecal occult blood test once every 2 years)	☐	☐	☐	☐
13. Adopt safe sex behavior(abstinence, single sex partner, use condom)	☐	☐	☐	☐
14. Use oral contraceptives	☐	☐	☐	☐

(B) Plan and Reason to change Health behavior

The original version is in Chinese and translated by authors

Note: During the **past 4 weeks**, how MUCH plan of health behavior that you have done in your impression?

15. How MUCH plan of health behavior change that you have done?

**No need to change (0)**: No idea of changing health behaviors at all.<br>**Consider to change (1):** There is an idea to change healthy behavior, but it hasn't started yet.<br>**Occasional changes (2)**: I will change my health behavior when I think about it, but there is no regularity.<br>**Has changed (3)**: My health behavior has been changed, but the change has not been more than six months.<br>**Maintain the change(4)**: I have changed my health behavior and it has changed for more than six months<br>**Complete the change(5)**: I am absolutely sure that will not return to the state of healthy behavior before the change	0 **No need to change**	1 **Consider to change**	2 **Occasional changes**	3 **Has changed**	4 **Maintain the change**	5 **Complete the change**
(e.g.) Plan of running			√			
1. Body weight control						
2. Fat intake						
3. Processed meat intake (bacon, sausage, ham, bacon, jerky, etc.)						
4. fruits and vegetables intake						
5. Physical activity or exercise						
6. Alcohol intake						
7. Smoking						
8. Reject second-hand smoke						
9. Pap smear						
10. Breast Screening						
11. Oral cancer screening						
12. Colorectal cancer screening						
13. Safe sex behavior						
14. Receive HPV vaccine						
15. Oral contraceptives used						
16. Other(please Specify) ____________________						

16. The following reasons to consider changing healthy behaviors, which are meaningful to you?

	Not at all important	Not much important	Neutral	Somewhat important	Very important
1. Practice healthy behaviors makes me healthier					
2. Taking responsibility for my health makes me more confident					
3. I can eat healthier and more nutritious					
4. Weight control can make me healthier					
5. Eating less high-fat foods can make me healthier					
6. Eating less processed meat can make me healthier					
7. Eat more fruits and vegetables can make me healthier					
8. At least 150 minutes of moderate or 75 minutes of strenuous activity per week can make me healthier					
9. Controlling the amount of smoking can make me healthier					
10. Refusing to smoke secondhand smoke can make me healthier					
11. Controlling alcohol intake can make me healthier					
12. Cancer screening can protect me					
13. HPV vaccine can protect me					
14. Pap smears are important for women					
15. Doing a Pap smear can detect and treat cervical cancer early					
16. Other(please Specify) ____________________________					

17. The following reasons for **not changing** healthy behaviors, which are meaningful to you?

	Not at all important	Not much important	Neutral	Somewhat important	Very important
1. No time due to work or household duties					
2. I am healthy, so I don' t need to change.					
3. I'm embarrassed and afraid that others will say what I do.					
4. Controlling weight is difficult for me					
5. It is difficult for me to eat less high-fat foods					
6. It is difficult for me to eat less processed meat					
7. It is difficult for me to eat more fruits and vegetables					
8. At least 150 minutes of moderate or 75 minutes of strenuous activity per week is difficult for me					
9. 9. It is difficult for me to stop smoking					
10. It is difficult for me to refuse secondhand smoke					
11. It is difficult for me to control the amount of drink					
12. Cancer screening is difficult for me					
13. HPV vaccine is not safe					
14. HPV vaccine is too expensive					
15. I don' t know where to get t HPV vaccine					
16. The male doctor performing Pap smear makes me feel very embarrassed					
17. Still looking for a doctor I can trust					
18. I don't have transportation to visit a doctor					
19. Time-consuming of visiting a doctor is too long for me					
20. It is inconvenient for me to get Pap smears					
21. Worried about bad Pap smear results					
22. Afraid need to do more invasive examinations (colposcopy, biopsy)					
23. Other(please Specify)____________________________					

(C)Self-efficacy of health behavior

This part is mainly to <u>**assess your ability**</u> to practice different healthy behaviors in your daily life. It has <u>**nothing to do with your actual practice**</u> of health behavior.

Completely unsure (0) of it (0%)
Little certainty (1) that it can be done (25%)
Half certainty (2) (50%)
Most of them (3) can do it (75%)
Can do (4) it (100%)

	completely unsure 0 %	little certainty 25%	half certainty 50 %	most of them 75 %	most of them 100 %
1. I can do at least 150 minutes of moderate physical activity (brisk walking, climbing, car washing, mopping, fitness exercises, etc.) or 75 minutes of strenuous physical activity (running, climbing stairs, playing ball, etc.) every week	☐	☐	☐	☐	☐
2. I can eat less fatty foods	☐	☐	☐	☐	☐
3. I can eat less processed meat	☐	☐	☐	☐	☐
4. I can eat more fruits and vegetables	☐	☐	☐	☐	☐
5. I can count my ideal weight and avoid mild obesity (BMI <27)	☐	☐	☐	☐	☐
6. I can drink less than 2 servings a day (1 serving:1 can of beer, 1 goblet of red wine or mixed wine, 1 cup of 50cc whiskey)	☐	☐	☐	☐	☐
7. I can stop smoking every day.	☐	☐	☐	☐	☐
8. I can protect myself from second-hand smoke	☐	☐	☐	☐	☐
9. I can do the health examination on time by my own schedule	☐	☐	☐	☐	☐
10. I can get a Pap smear once a year	☐	☐	☐	☐	☐

(D) Cervical cancer literacy

This part is to understand your awareness of cervical cancer. Please answer according to the actual situation (Some of them are reverse questions, not all of them are correct)	1 Right	2 wrong	3 unsure
1. Cervical cancer is a preventable cancer	☐	☐	☐
2. After menopause, it is not easy to get cervical cancer.	☐	☐	☐
3. Pap smear is a good way to screen for cervical cancer.	☐	☐	☐
4. If you have symptoms such as abnormal bleeding, vaginal discharge of odorous substances, uncomfortable sexual intercourse, etc., you can go to the hospital for a Pap smear test.	☐	☐	☐
5. Cryotherapy and partial cervical excision are the common treatments for cervical precancerous lesions.	☐	☐	☐

The original version is in Chinese and translated by authors

6. It means that you have cervical cancer when the result is low-grade cervical lesions (CIN 1). ☐ ☐ ☐
7. It means that you have cervical cancer when the abnormal result of Pap smear and HPV is positive. ☐ ☐ ☐
8. When you get an abnormal Pap smears or precancerous lesions result, you can discussed with your doctors about the treatment type based on your age and fertility planning. ☐ ☐ ☐
9. My doctor' s advice is the best and professional. I only need to do is by his/her order. ☐ ☐ ☐
10. I can get more examination or treatment information from other medical professionals, the Internet, medical websites, health magazines, books, TV programs, etc., and discuss the best method with the doctor. ☐ ☐ ☐

(F) QLQ30+CX24

During the **past 4 weeks**, how do you feel about yourself?	1 Not at all	2 A little	3 Quite a bit	4 Very much	Coding Code
1. Have you feel physically less attractive?	☐	☐	☐	☐	☐
2. Have you feel less feminine?	☐	☐	☐	☐	☐
3. Have you feel dissatisfied with your body?	☐	☐	☐	☐	☐
4. Have you worried that sex would painful?	☐	☐	☐	☐	☐
5. Have you been sexually activity?<br>☐0. No<br>☐1. Yes(Please answer question 6)					
6. Have you feel sexual discomfort during sexual intercourse?<br>☐0. No<br>☐1. Yes ☐2. vagina dry ☐3. vagina short ☐4. vagina tight ☐5.pain					

(G)DT emotion status

1. During the past week, answer and circle the emotion status based on your feeling e.g.

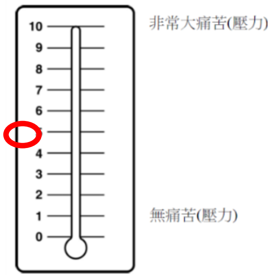

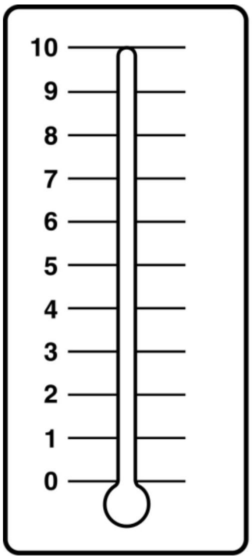

Very painful (pressure)

No pain (pressure)

(H) Health status

1. Please draw a "dot" on the scale on the left to show your health status today.

100= Self-evaluate the best health status

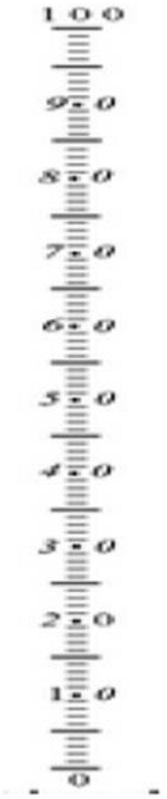

0= Self-evaluate the worst health status
